# Publisher Correction: Real space manifestations of coherent screening in atomic scale Kondo lattices

**DOI:** 10.1038/s41467-021-26341-5

**Published:** 2021-10-13

**Authors:** María Moro-Lagares, Richard Korytár, Marten Piantek, Roberto Robles, Nicolás Lorente, Jose I. Pascual, M. Ricardo Ibarra, David Serrate

**Affiliations:** 1 Laboratorio de Microscopias Avanzadas, Instituto de Nanociencia de Aragón, University of Zaragoza, E-50018 Zaragoza, Spain; 2 Institute of Physics, Academy of Sciences, Prague, 16200 Czech Republic; 3 Regional Centre of Advanced Technologies and Materials, Faculty of Science, Department of Physical Chemistry, Palacky University, Olomouc, 78371 Czech Republic; 4 Department of Condensed Matter Physics, Faculty of Mathematics and Physics, Charles University, 121 16 Prague 2, Czech Republic; 5 Dpto.Física Materia Condensada, University of Zaragoza, E-50009 Zaragoza, Spain; 6 Catalan Institute of Nanoscience and Nanotechnology (ICN2), CSIC and BIST, Campus UAB, Bellaterra, 08193 Barcelona, Spain; 7 Centro de Física de Materiales CFM/MPC (CSIC-UPV/EHU), 20018 Donostia-San Sebastián, Spain; 8 Donostia International Physics Center (DIPC), 20018 Donostia-San Sebastian, Spain; 9 CIC NanoGUNE, E-20018 Donostia-San Sebastián, Spain; 10 IKERBASQUE, Basque Foundation for Science, E-48011 Bilbao, Spain; 11 Instituto de Ciencia de Materiales de Aragón, CSIC - Universidad de Zaragoza, 50009 Zaragoza, Spain

**Keywords:** Magnetic properties and materials, Electronic properties and materials, Surfaces, interfaces and thin films

Correction to: *Nature Communications* 10.1038/s41467-019-10103-5, published online 17 May 2019.

The original HTML version of this Article was updated shortly after publication because the previous HTML version linked to an incorrect Transparent Peer Review file. The correct Peer Review file has now been linked.

