# Supplementary material for: Real space manifestations of coherent screening in atomic scale Kondo lattices
Source: Nat Commun. 2019 May 17;10:2211. doi: 10.1038/s41467-019-10103-5 (PMC6525169; doi:10.1038/s41467-019-10103-5)
Supplement: Supplementary file 1 — Supplementary Information [file 41467_2019_10103_MOESM1_ESM.pdf]

Supplementary information for: Real space manifestations of coherent screening in atomic scale Kondo lattices

María Moro-Lagares,[1,2,3] Richard Korytár,[4] Marten Piantek,[1,5] Roberto Robles,[6] Nicolás Lorente,[7,8] Jose I. Pascual,[1,9,10] M. Ricardo Ibarra,[1,5] and David Serrate[1,5,11,*]

[1]*Laboratorio de Microscopias Avanzadas, Instituto de Nanociencia de Aragón, University of Zaragoza, E-50018 Zaragoza, Spain*

[2]*Institute of Physics, Academy of Sciences, Prague, Czech Republic*

[3]*Regional Centre of Advanced Technologies and Materials, Faculty of Science, Department of Physical Chemistry, Palacky University,Olomouc, Czech Republic*

[4]*Department of Condensed Matter Physics, Faculty of Mathematics and Physics, Charles University, 121 16 Prague 2, Czech Republic*

[5]*Dpto.Física Materia Condensada, University of Zaragoza, E-50009 Zaragoza, Spain*

[6]*Catalan Institute of Nanoscience and Nanotechnology (ICN2), CSIC and BIST, Campus UAB,Bellaterra, 08193 Barcelona, Spain*

[7]*Centro de Física de Materiales CFM/MPC (CSIC-UPV/EHU), 20018 Donostia-San Sebastián, Spain*

[8]*Donostia International Physics Center (DIPC), 20018 Donostia-San Sebastian, Spain*

[9]*CIC NanoGUNE, E-20018 Donostia-San Sebastián, Spain*

[10]*IKERBASQUE, Basque Foundation for Science, E-48011 Bilbao, Spain*

[11]*Instituto de Ciencia de Materiales de Aragón, CSIC - Universidad de Zaragoza, 50009 Zaragoza, Spain*

(Dated: April 9, 2019)

SUPPLEMENTARY NOTE 1.- MODELLING AND IMAGING OF THE KONDO RESONANCE

Co atoms are imaged as protrusions with 95 pm height, dispersed over the Ag(111) (Supplementary Figure 1b). Energy resolved dI/dV spectra are, in general, proportional to the local density of states (LDOS) at the tip position. In particular, the LDOS of individual Co impurities over the metallic Ag(111) surface, hereafter ρ_S, exhibit a zero bias feature (ZBF) due to

* serrate@unizar.es

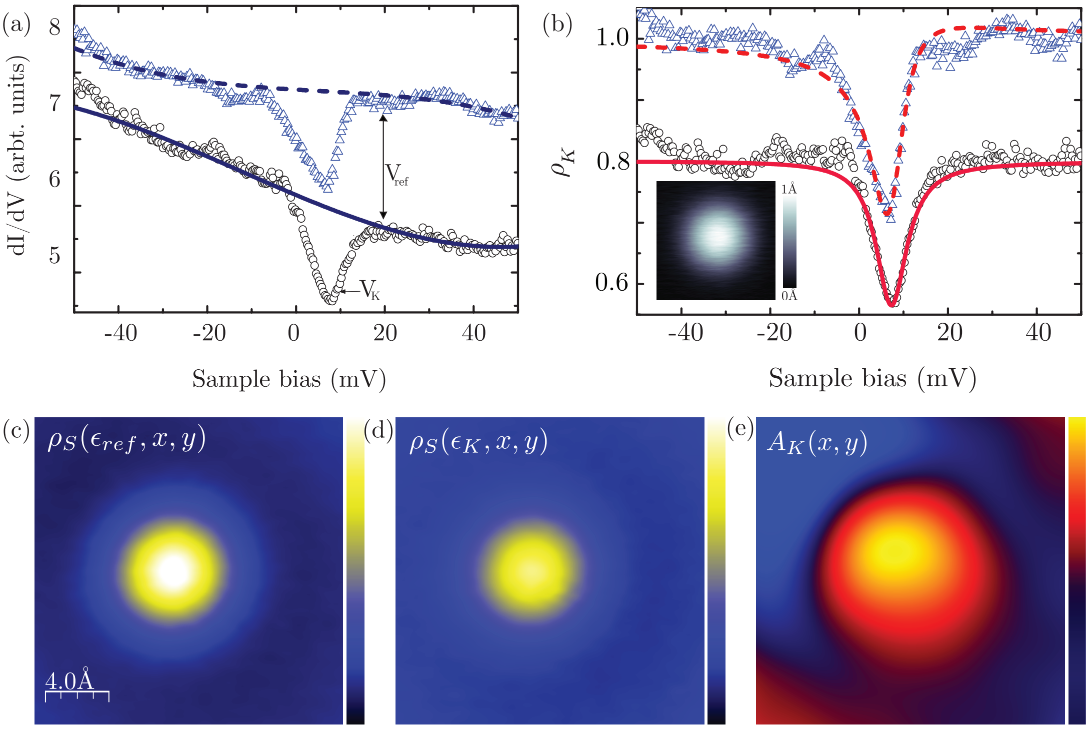

SUPPLEMENTARY FIGURE 1. (a) Exemplary raw $dI/dV(\rho_S(eV))$ showing the Kondo ZBF at the center of two Co atoms (circles and triangles) and $\rho_0(eV)$ background estimation (lines). (b) Fits of the resulting $\rho_K(eV)$ (circles and triangles) to Supplementary Equation 2 yielding $\Gamma_0/2$=4.3 ±0.3 meV, $q = 0.02 \pm 0.02$ and $\epsilon_0 = 7.1 \pm 0.2$ meV (solid line) and $\Gamma_0/2$=4.4 ±0.2 meV, $q = 0.28 \pm 0.02$ and $\epsilon_0 = 7.4 \pm 0.2$ meV (dashed line). Inset:typical constant current topography of the Co atom. Datasets in (a) and (b) have been vertically offseted for clarity. (c,d) Constant height dI/dV maps at V_{ref} ($\rho_S(eV_{ref})$) and V_K ($\rho_S(eV_K)$). (e) Kondo amplitude map ($A_K(x,y)$) given by Supplementary Equation 4. STM parameters: feedback regulation at -100 mV and 40 pA, bias modulation of 0.5 mV rms for spectroscopy and 2 mV rms for imaging.

the Kondo resonance. The ZBF can be expressed as[1]:

$$\rho_S(\epsilon) = (1 - A_K)\rho_0(\epsilon) + \rho_0(\epsilon)A_K\mathcal{F}(\epsilon), \quad (1)$$

where ρ_0 is the impurity LDOS in absence of Kondo screening, $\epsilon = eV$, $-e$ the electron charge, V the sample bias and A_K the Kondo amplitude. The first term represents the fraction of the tunnelling current not coupled to the discrete impurity state (as term C in ref. [1]) and the second one describes the tunnelling current coupled to the Kondo resonance. $\mathcal{F}(\epsilon)$ is the Fano function with linewidth Γ_0[1, 2]:

$$\mathcal{F}(\epsilon) = \frac{(q+\xi)^2}{1+\xi^2} \text{ with } \xi = \frac{\epsilon - \epsilon_0}{\Gamma_0/2}$$

To obtain the contribution owing exclusively to the Kondo effect, $\rho_S(\epsilon)$ is normalised by $\rho_0(\epsilon)$:

$$\rho_K \equiv \rho_S/\rho_0 = [(1 - A_K) + A_K\mathcal{F}(\epsilon)] \tag{2}$$

Here ρ_0 is a smooth polynomial background of third order which, in a our fitting routine, overlaps ρ_S exclusively in the region where the Kondo contribution is negligible ($\mathcal{F}(\epsilon) \simeq 1$), *i.e.*, $\mid \epsilon - \epsilon_0 \mid \geq 2\Gamma_0$. On the other hand, ρ_k was fitted to Supplementary Equation 2 in the region with strong Kondo contribution, defined by $\mid \epsilon - \epsilon_0 \mid \leq 1.5\Gamma_0/2$. Supplementary Figure 1a depicts how to estimate the ρ_0 background from raw dI/dV spectra. Supplementary Figure 1b displays the resulting $\rho_K(\epsilon)$ together with the fits to Supplementary Equation 2. Finally, Supplementary Figure 2 shows examples of spurious tip contributions to the Kondo resonance of individual Co atoms, which are identified as replicas of the features found in Co dimers built by atomic manipulation. Our spectroscopy measurements of the ZBF are obtained with tips without such contributions in the region $\mid \epsilon - \epsilon_0 \mid \leq 1.5\Gamma_0/2$. A rather commonly observed feature is the peak at around -10 mV (tip 1 in Supplementary Figure 2). While the comparison with the spectra acquired with other tips (over the same atom) unambiguously shows that this feature is tip dependent, we can not rule out that it stems from interference among channels opened by changes in tunnelling matrix elements connecting tip states with different orbital symmetry and sample electrons participating in the many-body KS.

The Kondo resonance linewidth is commonly expressed at low temperatures ($k_BT \ll \Gamma_0/2$) in terms of the Kondo temperature as $2k_B\sqrt{(\pi T)^2 + 2T_K^2} = \Gamma_0$[3]. In average, we obtain $T_K = 38 \pm 7$ K, $q = 0.15 \pm 0.2$ and $\epsilon_0 = 6.5 \pm 1$ meV, with data dispersion mainly ascribed to the atom position on the surface[4]. All atomic structures assembled in this work were constructed starting from a single atom whose individual Kondo resonance is characterised beforehand.

The identification of the half width at half maximum of a Kondo resonance (here $\Gamma_0/2$) with T_K has been recently found incorrect in the weak coupling regime between the spin impurity and the host metal electrons[5], for which the Fano lineshape fails to describe the logarithmic cusp of the zero-bias region. In our case, however, the resonance shape and width remain exactly the same in the range $T = 1.1$ K to $T = 4.7$ K, as predicted by the Fermi-liquid approach for $T \ll T_K$[3]. Therefore, the system falls in the strong coupling regime at the working temperature, and the use of a Fano lineshape is well justified.

Spatially resolved maps of the Kondo resonance are usually investigated by dI/dV or

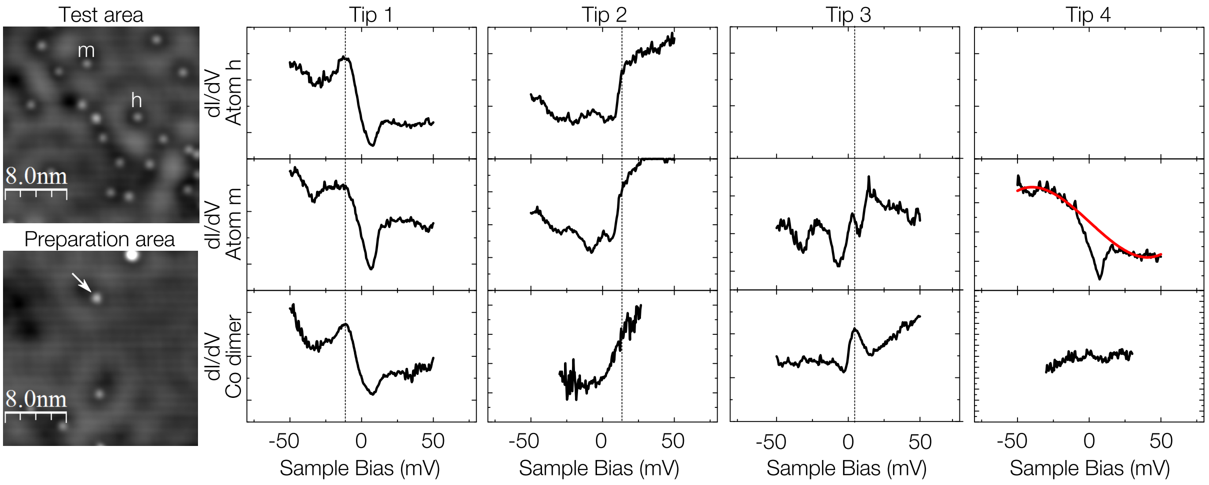

SUPPLEMENTARY FIGURE 2. Tip preparation and calibration for the characterization of Fano resonances. The raw dI/dV spectra of two Co atoms (m, l) are shown in the top and middle row for different tips prepared by gently dipping (100-300 pm) into the surface. The spectra of single atoms is compared to the spectra of a Co dimer built (white arrow) by atomic manipulation (bottom row), which is known to be featureless around the Fermi level (see Supplementary Figure 5). In reference spectra taken over dimers or over the bare surface, the tip resonances give rise to a low bias structure which appears convoluted with the Fano resonance of individual atoms.

$\mathrm{d}^2I/\mathrm{d}V^2$ imaging in constant current mode at bias voltages where the Fano-like spectra exhibit singularities[6–9]. In this work, Kondo amplitude images have been calculated from constant height $\rho_S(eV, x, y)$ slices, as the ratio between the LDOS at a bias voltage without Kondo contribution (V_{ref}, see Supplementary Figure 1a,c) and another one at the position of the Kondo dip minimum (V_K in Supplementary Figure 1a,d). This method to determine $A_K(x, y)$ is based on the fact that $\mathcal{F}(\xi_m) = 0$, where $\xi_m = -q$ minimizing $\mathcal{F}(\xi)$. Correspondingly, the bias at which ρ_S reaches its minimum is $\epsilon_{min} = \epsilon_0 - q\Gamma_0/2$. In all our spectra $|q| < 0.25$, $\Gamma_0/2 \simeq 4.5$ meV and $\epsilon_0 \simeq 6.5$ meV. Therefore we can take $\epsilon_{min} \simeq \epsilon_0$ constant with an approximation better than 1 meV, which is lower than our energy resolution for imaging with lock-in modulation of 2 mV rms. On the other hand, away from the Kondo resonance at $\xi \gg 1$, $\mathcal{F}(\xi)$ approaches rapidly $\mathcal{F}(\infty) = 1$ and consequently ρ_S becomes flat for $\epsilon_{ref} \geq 17$ meV. Under these conditions, $\rho_S(\epsilon_{ref})$ carries the topographic information and the ratio between $\rho_S(\epsilon_{min})$ and $\rho_S(\epsilon_{ref})$ provides the spatially resolved Kondo amplitude (see Supplementary Equation 1, illustrated in Supplementary Figure 1e for the case of the individual atom):

$$A_K(x,y) = 1 - \frac{\rho_0(\epsilon_{min})}{\rho_0(\epsilon_{ref})}\frac{\rho_S(\epsilon_{min})}{\rho_S(\epsilon_{ref})} \equiv 1 - \Theta(\epsilon_{min}, \epsilon_{ref}, x, y)\frac{\rho_S(\epsilon_{min})}{\rho_S(\epsilon_{ref})} \quad (3)$$

$$A_K(x,y) - 1 \propto -\frac{\rho_S(\epsilon_{min}, x, y)}{\rho_S(\epsilon_{ref}, x, y)} \quad (4)$$

The proportionality becomes strictly equal if $\rho_0(\epsilon)$ can be considered constant between ϵ_K and ϵ_{ref}. In most cases, $\rho_0(\epsilon)$ varies a lot slower than $\rho_K(\epsilon)$ (see Supplementary Figures 1a,b), and we can take $\Theta \simeq 1$. However, care must be taken that Θ does not deviate significantly from unity in the region of interest (background LDOS ρ_0 should be as flat as possible), which gives rise to artifacts like the reddish corners in Supplementary Figure 1e. Nevertheless, Supplementary Equation 4 captures essentially the spatial distribution of A_K, which is non-zero in a circular region of 14 Å diameter centred at the atom.

The analytical model given in ref. 1, widely accepted to explain the ZBF as a Fano lineshape, allows us to interpret the physical meaning of A_K. Following the notation in that reference, we find the following correspondence between Supplementary Equation 2 and the model's physical magnitudes

$$\rho_0 = \frac{4e^2}{\hbar}\rho_{tip}\rho_{0S} \quad (5)$$

$$\rho_{0S} = \pi \sum_k |M_{kt}|^2 \delta(eV - \epsilon_k) \quad (6)$$

$$A_K = \frac{|B|^2}{\mathrm{Im}\sum_d(eV)}\frac{1}{\rho_{0S}} \quad (7)$$

With ρ_{0S} the bare unperturbed conduction band LDOS without convolution with the tip states. The first factor in Supplementary Equation 7 corresponds to the number of conduction electrons coupled to the impurity spins in a single band model[1]. Therefore, A_K is the fraction of conduction electrons participating in the Kondo screening process.

SUPPLEMENTARY NOTE 2.- INTERACTIONS AMONG Co ATOMS

When two Co atoms approach each other, their corresponding dI/dV spectra exhibit small differences. These differences can be ascribed to either the LDOS variations of the supporting surface[4] or the interaction between the atoms. We took special care of removing variations of the ZBFs due to inhomogeneities in the surface LDOS. In order to discern both contributions, we performed the following experiment.

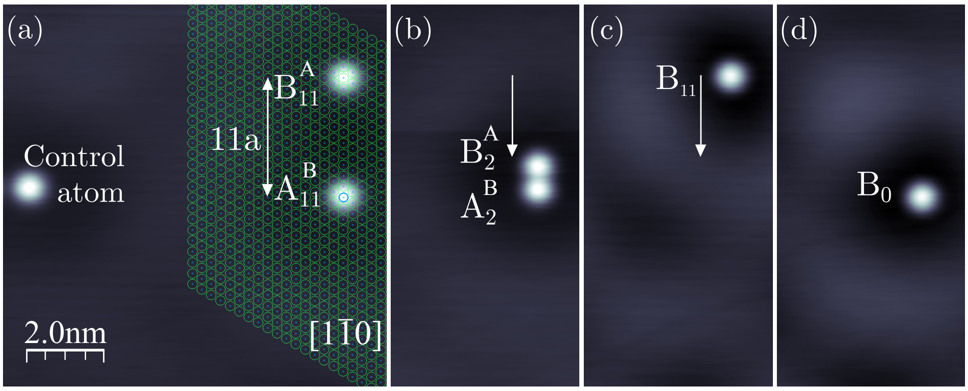

SUPPLEMENTARY FIGURE 3. Study of the interaction between two Co-atoms. a) Starting from two Co atoms at $d = 11a$, atom B is approached to atom A. b) When atoms are at $d = 2a$, atom A is moved far away from atom B. (c-d) Atom B is moved along the way back visiting the same atomic sites as before, but in absence of atom A. After each atomic manipulation, both atom B and atom A are characterized by constant height spectroscopy (feedback opened at regulation set point $V_0 = -20$ mV, $I_0 = 15$ pA over Ag surface). In addition, dI/dV of a control atom it is used to check the featureless density of states (DOS) of the tip after each manipulation event.

Starting from two Co atoms separated $d = 11a$ (Supplementary Figure 3a), by lateral manipulation, atom B is approached towards atom A in steps of one Ag(111) interatomic distance, a. After each atomic manipulation we measured ρ_S in both A and B atoms and in a third control atom far away. The control atom allows us checking that the tip LDOS remains unchanged after each manipulation. In this experiment, variations in ρ_S of atom B contain both the influence of the substrate position and the interaction with A. Atom A stays fixed and therefore its ρ_S is only affected by the relative distance to B. When B reaches $d = 2a$ (Supplementary Figure 3b), atom A is moved far away. Then, atom B is moved again along the previous path visiting the same atomic sites as before (Supplementary Figures 3c,d). Now, ρ_S variations in B are only contributed by the effect of the different substrate positions. Let δA_i^B be the change in A_K that atom B (at $d = i \times a$) produces in atom A (at position $i = 0$), and δB_i^A that of atom B (at $d = i \times a$) produced by atom A. At $d = 11a$, the two Co atoms are too far to interact and behave as isolated single atoms. Thus, in order to extract the relative variations of the Kondo amplitude, we normalised δA_i^B and δB_i^A to the A_K value of the respective Co atoms at $d = 11a$. By definition, they can be written as:

$$\frac{\delta A_i^B}{A_{11}^B} = \frac{A_i^B - A_{11}^B}{A_{11}^B} \tag{8}$$

$$\frac{\delta B_i^A}{B_{11}^A} = \frac{B_i^A - B_i}{B_{11}^A} \tag{9}$$

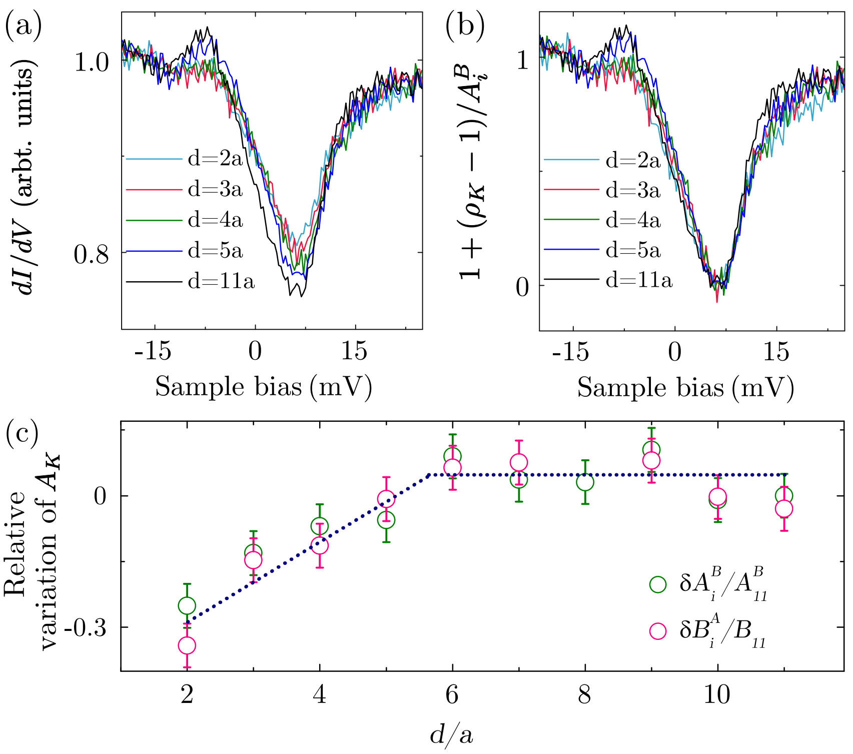

SUPPLEMENTARY FIGURE 4. (a) ρ_K of atom A with B at several distances showing the stepwise amplitude decrease. (b) Same data as in (a) but normalised by the value of A_i^B. The ZBFs collapse in one curve proving that the rest of the lineshape parameters (Γ_0, q and ϵ_0) are constant. (c) Relative variation of A_K in A and B versus interatomic distance, defined by Supplementary Equations 8-9. Error bars are SD resulting from the fit of the spectra to Supplementary Equation (1). STM parameters: feedback regulation at -20 mV and 15 pA,bias modulation for spectroscopy of 0.5 mV rms.

Where A_i^B is the Kondo amplitude of atom A when atom B is at $d = i \times a$, B_i^A that of atom B at $d = i \times a$ from A, and B_i that of atom B alone at $d = i \times a$ from the site previously occupied by A. These amplitudes are extracted from fits of individual spectra to Supplementary Equation 2 (Supplementary Figures 4a,b show some selected resonances). In this way, the expressions in Supplementary Equations 8 and 9 account for the Kondo amplitude variations owing exclusively to interactions between atoms. We do not observe any relevant changes in the Kondo temperature (Supplementary Figure 4b). However, there is a gradual decrease of the Kondo amplitude for $d \leq 5a$ (Supplementary Figure 4c).

Finally, we inspected the properties of a Co dimer at $d = a$. Supplementary Figures 5a-c show the fabrication of a Co compact dimer starting from two individual Co atoms. The compact dimer displays a featureless dI/dV spectra (leading to a flat ρ_k with $A_K = 0$, see Supplementary Figure 5d). In this situation, Co atomic orbitals can overlap, and direct exchange interaction overcomes the Kondo state biding energy, leading to a magnetic ground state incompatible with the Kondo effect[10–12]. This sudden disappearance of the Kondo resonance contrasts sharply with the smooth variations of A_K for $d \geq 2a$, indicating that different interaction mechanisms govern each distance range.

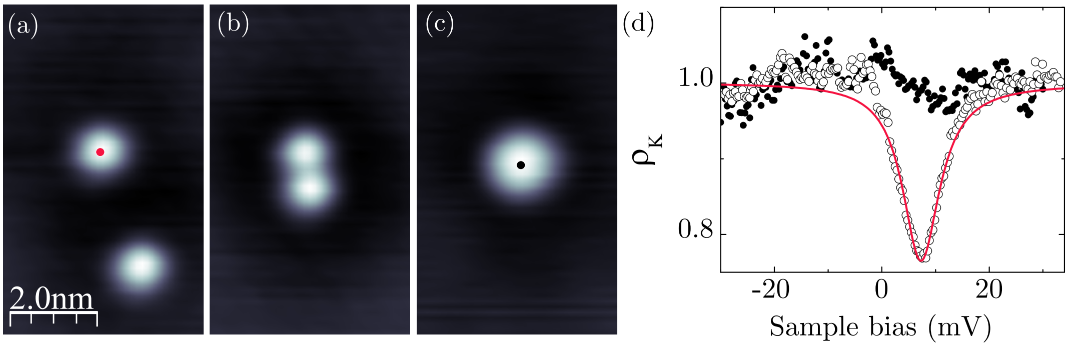

SUPPLEMENTARY FIGURE 5. Formation of a compact dimer:(a,b,c) STM topographs (-20 mV, 100 pA) of different steps of the atomic manipulation process leading to a compact dimer. (d) ZBF of the original Co atom (open circles), its corresponding Fano fit (red line) and the featureless resonance of the compact dimer (black dots).

Supplementary Figure 6 shows how the Kondo redistribution pattern does not take place if one of the atoms of the dimer is not Kondo screened, as in the case of the Ag-Co dimer.

Supplementary Figure 7 shows how the shape of the ZBF is maintained also in longer chains regardless of its final width and the construction stage. This allows us to disregard any other inter-impurity coupling that is not associated to Kondo fluctuations as detailed in Supplementary Note 4.

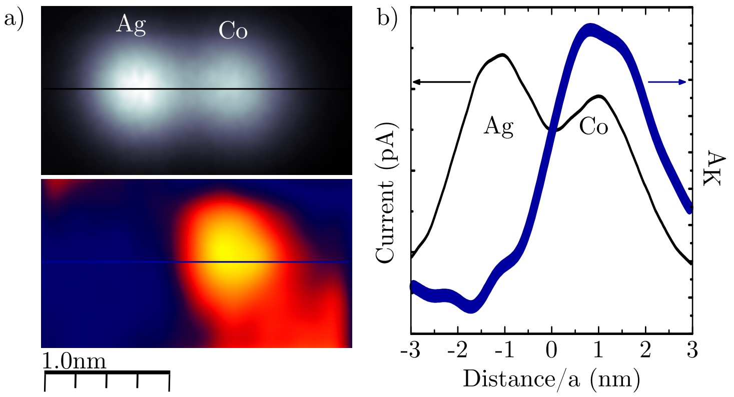

SUPPLEMENTARY FIGURE 6. (a) Simultaneous constant height images of the tunnelling current (top) and Kondo amplitude, A_K (bottom) of a Ag-Co dimer at $d = 2a$. (b) Horizontal line profile across the dimer of the current (thin black line) and A_K (thick blue line). Constant height regulation set point of -100 mV and 40 pA over the substrate, lock-in modulation 2 mV rms, $V_{ref} = 20$ mV.

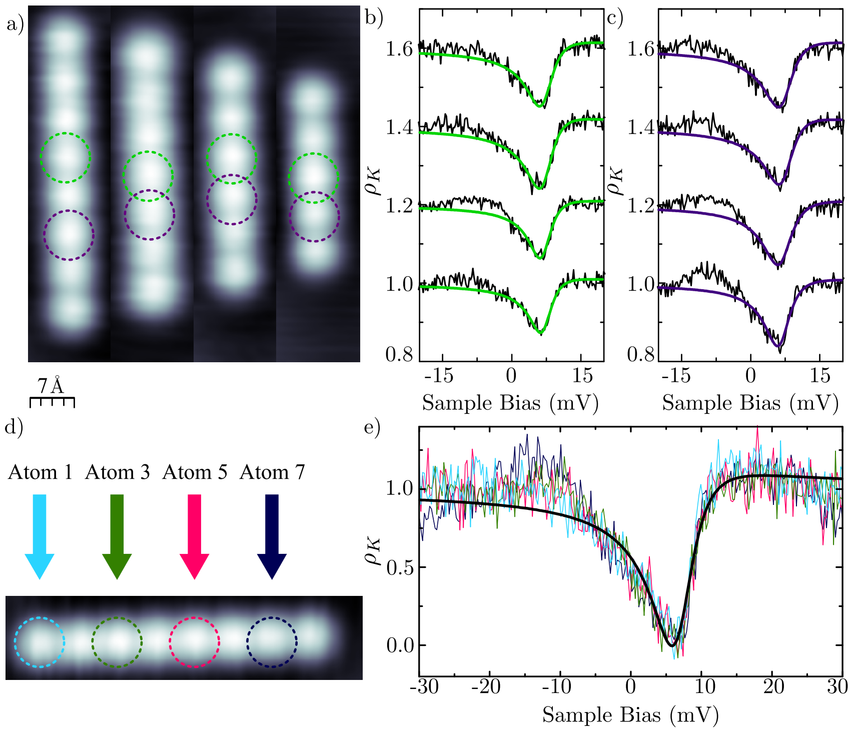

SUPPLEMENTARY FIGURE 7. (a) Construction of N=5,6,7 and 8 chains with $d = 2a$. The dashed circles indicate the same atom along the process. (b) and (c) Kondo resonances corresponding to the marked circles in (a) in increasing order of length chain from bottom to top, offseted by clarity. Lines are fits to Supplementary Equation 2 (d) Final state of the constructed chain. (e) Kondo resonances of the atoms highlighted in panel d. STM parameters: feedback regulation at -100 mV and 40 pA, bias modulation for spectroscopy of 0.5 mV rms. All ZBF in spectroscopy can be fitted with the following set of parameters in Supplementary Equation 2: $q = 0.3 \pm 0.1$, $\Gamma_0 = 7.4 \pm 0.6$ meV and $\epsilon_0 = 6.8 \pm 0.5$ meV

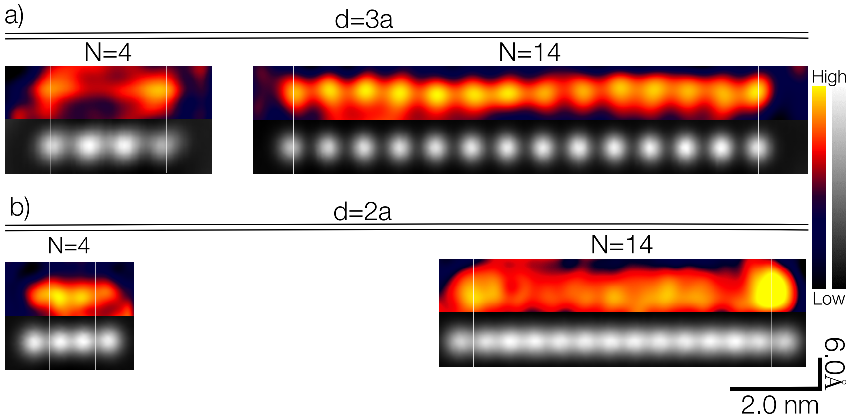

SUPPLEMENTARY FIGURE 8. Simultaneous constant height tunnelling current (grayscale) and Kondo amplitude A_K (red-yellow scale) maps of N Co atoms chains with spacing $d = 3a$ (a) and $d = 2a$ (b). These images together with the ones in Fig. 4 of the main text are used to build the experimental edge-lobe region in Fig. 5. The scale bar applies to the entire figure. All tunneling current images (greyscale) were acquired at $V_{ref} = 17 - 20$ mV sample bias.

SUPPLEMENTARY NOTE 3.- SELF-CONSISTENT PERTURBATION THEORY FOR THE MULTI-IMPURITY ANDERSON MODEL

Our aim is to approximately treat the multi-impurity Anderson Hamiltonian,

$$\hat{H} = \hat{H}_{\rm i} + \hat{H}_{\rm c} + \hat{V} \tag{10}$$

$$\hat{H}_{\rm c} = \sum_{k\sigma} \epsilon_k \hat{c}^\dagger_{k\sigma} \hat{c}_{k\sigma} \tag{11}$$

$$\hat{H}_{\rm i} = \epsilon_{\rm d} \sum_{i=1}^{N} \sum_{\sigma} \hat{d}^\dagger_{i\sigma} \hat{d}_{i\sigma} + U \sum_{i=1}^{N} \hat{d}^\dagger_{i\uparrow} \hat{d}_{i\uparrow} \hat{d}^\dagger_{i\downarrow} \hat{d}_{i\downarrow} \tag{12}$$

$$\hat{V} = \sum_{k\sigma} \sum_{i=1}^{N} V_{ki} \hat{c}^\dagger_{k\sigma} \hat{d}_{i\sigma} + \text{ h.c.} \tag{13}$$

which involves two sub-spaces: the conduction electrons governed by a bilinear Hamiltonian $\hat{H}_{\rm c}$ (a Gaussian fermionic bath, operators $\{\hat{c}^\dagger_{k\sigma}, \hat{c}_{k\sigma}\}$) and the subspace of the interacting impurities with fermionic operators $\{\hat{d}^\dagger_{i\sigma}, \hat{d}_{i\sigma}\}$. A self-consistent diagrammatic perturbation theory for these types of systems was developed by Keiter, Morandi, Kuramoto, Grewe and others and successively applied to single-impurity systems [13–15]. These techniques can be straightforwardly generalized to multiple impurities.[16]

First, we discuss the configurations in the impurity space. If on site impurity state energy $\epsilon_{\rm d} < 0$ and $|\epsilon_{\rm d}| < U$, the lowest-energy configuration (charge state) in the impurity space is

the half-filling, having a single electron per impurity. This configuration involves many-electron states of the form

$$\hat{d}^{\dagger}_{1\tau_1}\hat{d}^{\dagger}_{2\tau_2}\dots\hat{d}^{\dagger}_{N\tau_N}|\rangle =: |\tau_1\dots\tau_N\rangle = |\beta\rangle \tag{14}$$

where $|\rangle$ is the impurity vacuum (free from electrons). These states can be indexed by the spin projections on each impurity $\tau_1\dots\tau_N$. On the right hand we introduce two shorthand notations.

We shall assume that the lowest excited configuration is the half-filling with a single hole. Specifically, the states of the form

$$(-1)^{n+1}\hat{d}^{\dagger}_{1\sigma_1}\dots\hat{d}^{\dagger}_{n-1\sigma_{n-1}}\hat{d}^{\dagger}_{n+1\sigma_{n+1}}\dots\hat{d}^{\dagger}_{N\sigma_N} := |\alpha\rangle := |\sigma_1\dots\sigma_{n-1}0\sigma_{n+1}\dots\sigma_N\rangle \tag{15}$$

having a hole in the impurity orbital n. Again, the r.h.s. shows two shorthand notations. Higher-energy configurations correspond to other charge states of the impurities: double occupancies or multiple holes, for example.

Now we proceed to make a truncation approximation by restricting ourselves in the following calculation to these two impurity configurations, $|\alpha\rangle$ and $|\beta\rangle$. This approximation is equivalent to the commonly employed $U = \infty$ limit and furthermore discards certain multiple-hole correlations. We note that a common step in other works is to introduce Schrieffer-Wolff transformation of the Anderson Hamiltonian. The latter transformation involves a single virtual hole as well (if $U = \infty$). Thus, it would imply the same truncation as adopted in the present work.

The (bare) impurity Hamiltonian can be rewritten in the truncated form

$$\hat{H}_{\rm i} \;\mapsto\; \epsilon_{\rm d}\sum_{\beta}|\beta\rangle\langle\beta|. \tag{16}$$

The hybridization $\hat{V}$, upon truncation, becomes

$$\hat{V} = \sum_{k\sigma}\sum_{\alpha\beta} V_{k\sigma}(\alpha|\beta)\cdot\hat{c}^{\dagger}_{k\sigma}|\alpha\rangle\langle\beta| \; + V_{k\sigma}(\beta|\alpha)\cdot|\beta\rangle\langle\alpha|\hat{c}_{k\sigma}. \tag{17}$$

The canonical operators $\hat{d}_{i\sigma}, \hat{d}^{\dagger}_{i\sigma}$ are replaced by the Hubbard operators $|\alpha\rangle\langle\beta|$ and $|\beta\rangle\langle\alpha|$, respectively, and the hybridization matrix elements are expressed as

$$V_{k\sigma}(\alpha|\beta) = V_{kn}\delta_{\tau_1\sigma_1}\dots\delta_{\tau_{n-1}\sigma_{n-1}}\delta_{\tau_n\sigma}\delta_{\tau_{n+1}\sigma_{n+1}}\dots\delta_{\tau_N\sigma_N}, \qquad V_{k\sigma}(\beta|\alpha) = V^{*}_{k\sigma}(\alpha|\beta) \tag{18}$$

where n indexes the hole.

Following Ref. [17] we introduce resolvents $R_{\alpha\alpha'}(\omega)$ and $R_{\beta\beta'}(\omega)$ for the two configurations. The effects of the hybridization with conduction electrons enter via self-energies in the usual way.

Notice, that the off-diagonal terms of $R_{\beta\beta'}(\omega)$ are activated by the dynamic RKKY couplings generated by the conduction electrons. Moreover, certain off-diagonal terms of $R_{\alpha\alpha'}$ with holes at different locations correspond to substrate-induced inter-impurity hybridization. We focus on the regime of negligible RKKY interactions, specifically, when the energy difference between different spin states is smaller than the width of the Kondo resonance (see Supplementary Note 4). In this regime, we may restrict the resolvent $R_{\beta\beta'}$ to be diagonal in spin space,

$$R_{\beta\beta'}(\omega) = R^{(N)}(\omega) \times \delta_{\tau_1\tau_1'} \ldots \delta_{\tau_N\tau_N'} \tag{19}$$

and, similarly, suppress RKKY coupling in $R_{\alpha\alpha'}$, and retain only the spin-diagonal part of it, which we shall denote by $R_{ij}^{(N-1)}(\omega)$, where the superscript denotes the charge state and the subscript indices refer to impurities $i, j = 1, \ldots N$. This dependence has to be kept, because of the substrate-induced hybridization, that is expressed here via a coupling of holes at two different locations.

The hybridization self-energies $\Sigma^{(N)}(\omega), \Sigma_{ij}^{(N-1)}(\omega)$ are defined by the relations $R^{(N)}(\omega) = \left[\omega - \epsilon_{\rm d} - \Sigma^{(N)}(\omega)\right]^{-1}$ and $R_{ij}^{(N-1)}(\omega)$ equal to the inverse matrix of $\omega\delta_{ij} - \Sigma_{ij}^{(N-1)}(\omega)$. The self-energies can be calculated diagrammatically [17]. Neglecting vertex-corrections, the self-energies can be determined by the self-consistent equations

$$\Sigma^{(N)}(\omega) = \sum_{ij} \int n_{\rm F}(-\omega')\Gamma_{ij}(\omega')R_{ji}^{(N-1)}(\omega - \omega'){\rm d}\omega' \tag{20}$$

$$\Sigma_{ij}^{(N-1)}(\omega) = (1 + \delta_{ij}) \int n_{\rm F}(\omega')\Gamma_{ij}(\omega')R^{(N)}(\omega + \omega'){\rm d}\omega' \tag{21}$$

where $n_{\rm F}$ is the Fermi-Dirac function and $\Gamma_{ij}(\omega) = \sum_k V_{ki}^* V_{kj}\delta(\omega - \epsilon_k)$ is the anti-Hermitian part of the single-particle hybridization self-energy. The factor $(1 - \delta_{ij})$ expresses the fact, that the "on-site" term with a single hole is renormalized by two channels (corresponding to two spin directions) and the off-site terms are renormalized by only one channel, because the conduction band is spin-conserving. The pair of self-consistent equations is formally similar to the second-order self-consistent perturbation theory for a multi-orbital Anderson Hamiltonian. This is a consequence of the truncation, which restricts the problem to two charge states (configurations). The vertex corrections are negligible in the mathematical limit $N \to \infty$ [18].

The Feynman rules [17] lead to an expression for the physical d-electron Green's function of the impurities

$$G_{ij}(\omega) = \frac{2^{N-1}}{Z_{\rm i}} \int {\rm d}\omega' e^{-\beta\omega'} \left[A_{ij}^{(N-1)}(\omega')R^{(N)}(\omega + \omega') - R_{ji}^{(N-1)\star}(\omega' - \omega)A^{(N)}(\omega')\right] \tag{22}$$

The inverse temperature is denoted by β, Z_i is the partition function of the impurity subspace and we have introduced the spectral densities $A^{(N)}(\omega) = -\mathrm{Im}R^{(N)}(\omega)/\pi$ and $A_{ij}^{(N-1)}(\omega) = -\big[R_{ij}^{(N-1)}(\omega) - R_{ji}^{(N-1)\star}(\omega)\big]/2\pi i$ (all resolvents and propagators are retarded). Note that the Green's function of the impurities adopts a non-local form. The physical Green's function has to be evaluated numerically by the technique of defect propagators [19].

Now we turn our attention to the hybridisation function $\Gamma_{ij}(\omega),\ i,j = 1,\dots N$, which fully characterizes the substrate's effect on the impurity system. In this section we derive analytical expressions for a homogeneous electron gas in two and three dimensions. Due to homogeneity we may place the impurity j at a coordinate origin and assign the position vector $\mathbf{R}$ to impurity i.

We assume that the hopping element between the impurity at $\mathbf{R}$ and a plane wave has the form $V_{\mathbf{k}i} \equiv V_{\mathbf{kR}} \equiv \langle\mathbf{k}|\hat{H}|\mathbf{R}\rangle = ve^{-i\mathbf{k}\cdot\mathbf{R}}\sqrt{V_\mathrm{i}/V}$ adequate for point-like impurities ($V_\mathrm{i} \ll \lambda_\mathrm{F}^d$). The V and V_i are d-dimensional volumes of the system and the impurity and v is the scale-independent hopping matrix element. The off-diagonal matrix element of the hybridization function of two impurities at $\mathbf{R}$ and $\mathbf{0}$ reads

$$\Gamma_{\mathbf{R0}}(\omega) = \sum_{\mathbf{k}} V_{\mathbf{kR}}^{*} V_{\mathbf{k0}} \delta(\omega - \epsilon_{\mathbf{k}}) = v^2 V_\mathrm{i} \int \frac{\mathrm{d}^d\mathbf{k}}{(2\pi)^d} e^{i\mathbf{k}\cdot\mathbf{R}} \delta(\omega - \epsilon_{\mathbf{k}}) \tag{23}$$

($\hbar = 1$). The last expression corresponds to the limit $V \to \infty$. Since the Kondo physics is determined by the behavior of $\Gamma_{\mathbf{R0}}(\omega)$ at the Fermi level, we simplify the energy dependence of the hybridization function by the rectangular form determined by the cutoffs $\pm E_0$, as commonly done in the impurity models. The integral can be evaluated to

$$\Gamma_{\mathbf{R0}}(\omega) = \Gamma_0\ J_0\left(k_\mathrm{F}R\right) \cdot \Theta(E_0^2 - \omega^2) \qquad D = 2 \tag{24a}$$

$$\Gamma_{\mathbf{R0}}(\omega) = \Gamma_0\ \frac{\sin\left(k_\mathrm{F}R\right)}{k_\mathrm{F}R} \cdot \Theta(E_0^2 - \omega^2) \qquad D = 3. \tag{24b}$$

The Fermi wave-vector has been denoted by k_F and Γ_0 includes all pre-factors. J_0 is the zeroeth order Bessel function.

Finally, we comment briefly on the role of the frequency dependence. Formally, the frequency dependence would be established by replacing the constant k_F by $k(\omega)$ in the arguments of sin or J_0 inside Supplementary Equations 24. Consequently, for large but fixed R the function $\Gamma_{\mathbf{R0}}(\omega)$ oscillates in the frequency space (within the bandwidth). When the period of these oscillation exceeds the Kondo scale $k_\mathrm{B}T_\mathrm{K}$, the long-range hybridization via $\Gamma_{\mathbf{R0}}(\omega)$ becomes ineffective. This happens at impurity separations $R \approx v_\mathrm{F}/k_\mathrm{B}T_\mathrm{K}$, commonly known as the range of the Kondo cloud.

I. SUPPLEMENTARY NOTE 4.- CALCULATION OF THE RKKY COUPLING

In this Note we consider the exchange coupling between impurity spins mediated by the conduction electrons, within the MIAM. Specifically, an effective interaction of the form $\hat{H}_{\mathrm{RKKY}} = \frac{1}{2}\sum_{i\neq j} I(\mathbf{R}_i - \mathbf{R}_j)\hat{\mathbf{S}}_i \cdot \hat{\mathbf{S}}_j$ involving spin operators $\hat{\mathbf{S}}_j$ of the impurities is assumed. The $\hat{H}_{\mathrm{RKKY}}$ is commonly denoted as RKKY coupling [20]. The leading contributions to the exchange constant $I(\mathbf{R})$ are of fourth order in the hybridization elements V_{ki}.

The effective description of the impurities involving only the spin degrees of freedom is achieved by the means of a Schrieffer-Wolf transformation (SW^n) to a given order n in V_{ki}. The expression for the exchange constant $I(\mathbf{R})$ in the fourth order of the hybridization elements $V_{\mathbf{k}i}$ was given by Proetto and López [21]. It involves a term from SW^2 treated in second order and an RKKY-type term from SW^4. We assume the hybridization matrix elements have the spatial dependence given by $V_{kj} \equiv V_{k\mathbf{R}_j} = ve^{-i\mathbf{k}\cdot\mathbf{R}_j}\sqrt{V_{\mathrm{i}}/V}$ as in the SI Note 4C. The expression for the exchange then reads [21]

$$I(\mathbf{R}) = -v^4 V_{\mathrm{i}}^2 \sum_{\mathbf{q}} e^{i\mathbf{q}\cdot\mathbf{R}} \sum_{\mathbf{k}} \frac{1-n_{\mathbf{k}}}{(\epsilon_{\mathbf{k}}-\epsilon_{\mathrm{d}})^2} \left(\frac{n_{\mathbf{k}+\mathbf{q}}}{\epsilon_{\mathbf{k}+\mathbf{q}}-\epsilon_{\mathbf{k}}} + \frac{1-n_{\mathbf{k}+\mathbf{q}}}{\epsilon_{\mathbf{k}+\mathbf{q}}-\epsilon_{\mathrm{d}}} \right) \tag{25}$$

where $n_{\mathbf{k}} := n(\epsilon_{\mathbf{k}})$ is the Fermi-Dirac distribution. It will be convenient to express the formula in terms of the hybridization function as

$$I(\mathbf{R}) = -\iint \mathrm{d}\omega \mathrm{d}\omega' \; \Gamma_{\mathbf{0R}}(\omega)\Gamma_{\mathbf{R0}}(\omega') \frac{1-n(\omega)}{(\omega-\epsilon_{\mathrm{d}})^2} \left[\frac{n(\omega')}{\omega'-\omega} + \frac{1-n(\omega')}{\omega'-\epsilon_{\mathrm{d}}} \right]. \tag{26}$$

The long-range asymptotics of the Supplementary Equation 26 is a well-known oscillating power-law decay [22]. Here we focus on the short-range part determined by the condition $|\mathbf{R}| < \lambda_{\mathrm{F}}$. In this case, the frequency dependence of the hybridization function is weak and can be neglected (within the bandwidth). The remaining integral can be evaluated; with the small parameter $a = -\epsilon_{\mathrm{d}}/E_0 > 0$ we obtain

$$I(\mathbf{R}) = \frac{[\Gamma_{\mathbf{R0}}(0)]^2}{E_0} \left[-2\log(a) + 2\,(1-\log 2) + \frac{3}{2}a \right] + \mathcal{O}(a^2) \tag{27}$$

The first term is leading in the limit of small a. Interestingly, this term originates from both types of expressions: SW^2 and SW^4.

We compare the functional dependence of $I(\mathbf{R})$ on the parameters of the MIAM with the functional dependence of the single-impurity Kondo temperature, $k_{\mathrm{B}}T_{\mathrm{K}}^{(1)} = E_0 \exp(\epsilon_{\mathrm{d}}/2\Gamma)$ (infinite on-site repulsion). An upper estimate of $|I(\mathbf{R})|$ can be obtained by replacing $\Gamma_{\mathbf{R0}}(0)$ by Γ, the diagonal element. Let us fix $a = -\epsilon_{\mathrm{d}}/E_0$. Consequently, for infinitesimal Γ the RKKY

clearly prevails. Yet, upon increasing Γ there is a transition to the regime where the $k_{\mathrm{B}}T_{\mathrm{K}}^{(1)}$ dominates.

Finally, we comment that for the MIAM parametrization employed in the long chain calculation (see Methods) the RKKY scale is 30 times smaller than the single-impurity Kondo scale, justifying the omission of RKKY terms.

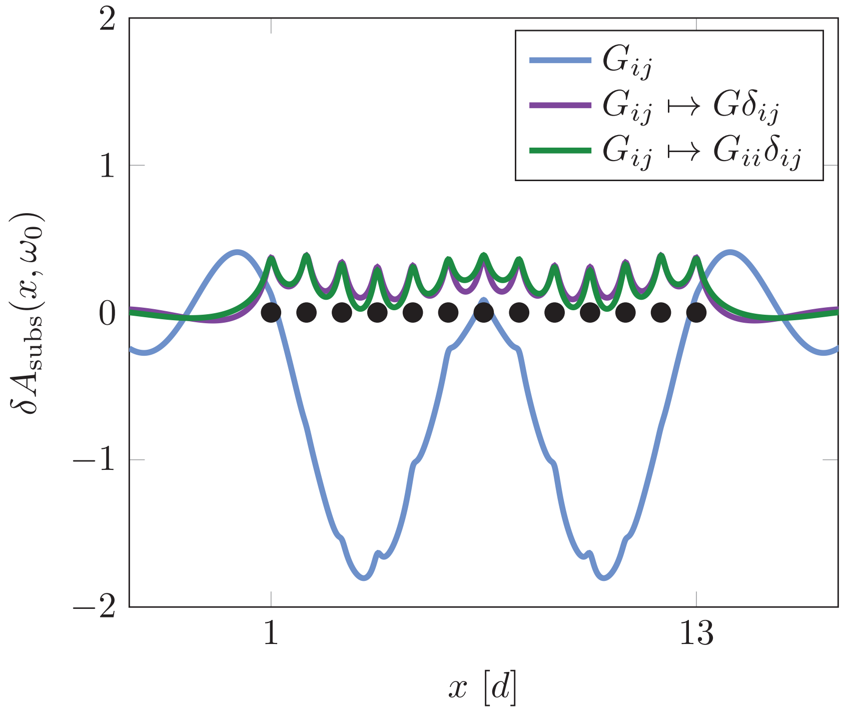

SUPPLEMENTARY FIGURE 9. $A_{\rm subs}$ change in the substrate LDoS due to a chain of 13 impurities at the frequency of the maximum of the Kondo peak, ω_0. The coordinate x is the axis parallel to the impurity chain. Black dots indicate the positions of the impurities. The blue line involves the full Green's matrix in the impurity space, G_{ij} (see Eq. (22)). The green line involves the G_{ij} with off-diagonal terms made zero. Additionally, the diagonal elements of G_{ij} are replaced by a constant (average) in the magenta line.

SUPPLEMENTARY REFERENCES

---

[1] V. Madhavan, W. Chen, T. Jamneala, M. F. Crommie, and N. S. Wingreen, Phys. Rev. B, **64**, 165412 (2001).

[2] V. Madhavan, W. Chen, T. Jamneala, M. F. Crommie, and N. S. Wingreen, Science, **280**, 567 (1998).

[3] K. Nagaoka, T. Jamneala, M. Grobis, and M. F. Crommie, Phys. Rev. Lett., **88**, 077205 (2002).

[4] M. Moro-Lagares, J. Fernández, P. Roura-Bas, M. R. Ibarra, A. A. Aligia, and D. Serrate, Phys. Rev. B, **97**, 235442 (2018).

[5] Y.-h. Zhang, S. Kahle, T. Herden, C. Stroh, M. Mayor, U. Schlickum, M. Ternes, P. Wahl, and K. Kern, Nat. Commun., **4**, 2110 (2013).

[6] Y. Jiang, Y. N. Zhang, J. X. Cao, R. Q. Wu, and W. Ho, Science, **333**, 324 (2011).

[7] A. DiLullo, S.-H. Chang, N. Baadji, K. Clark, J.-P. Klckner, M.-H. Prosenc, S. Sanvito, R. Wiesendanger, G. Hoffmann, and S.-W. Hla, Nano Letters, **12**, 3174 (2012).

[8] U. G. E. Perera, H. J. Kulik, V. Iancu, L. G. G. V. Dias da Silva, S. E. Ulloa, N. Marzari, and S.-W. Hla, Phys. Rev. Lett., **105**, 106601 (2010).

[9] A. Mugarza, C. Krull, R. Robles, S. Stepanow, G. Ceballos, and P. Gambardella, Nat. Commun., **2**, 490 (2011).

[10] C. Jayaprakash, H. R. Krishna-murthy, and J. W. Wilkins, Phys. Rev. Lett., **47**, 737 (1981).

[11] W. Chen, T. Jamneala, V. Madhavan, and M. F. Crommie, Phys. Rev. B, **60**, R8529 (1999).

[12] T. Jamneala, V. Madhavan, and M. F. Crommie, Phys. Rev. Lett., **87**, 256804 (2001).

[13] H. Keiter and G. Morandi, Physics Reports, **109**, 227 (1984), ISSN 0370-1573.

[14] Y. Kuramoto, Zeitschrift für Physik B Condensed Matter, **53**, 37 (1983).

[15] N. Grewe, Zeitschrift für Physik B Condensed Matter, **52**, 193 (1983).

[16] In principle, one has to work within the full configuration space of the impurities. Because the conduction electrons are Gaussian, Wick's theorem can be applied and perturbation theory in hybridization can be formulated diagrammatically.

[17] N. Bickers, Reviews of modern physics, **59**, 845 (1987).

[18] P. Coleman, Phys. Rev. B, **29**, 3035 (1984).

[19] J. Otsuki and Y. Kuramoto, Journal of the Physical Society of Japan, **75**, 064707 (2006).

[20] J. Stöhr and H. C. Siegmann, *Magnetism* (Springer, Berlin, Heidelberg., 2006).

[21] C. Proetto and A. López, Physical Review B, **24**, 3031 (1981).

[22] C. Proetto and A. López, Physical Review B, **25**, 7037 (1982).
